# Nutrient-adequate diets with the lowest greenhouse gas emissions or price are the least acceptable—insights from dietary optimisation modelling using the iOTA model^®^

**DOI:** 10.3389/fnut.2025.1596081

**Published:** 2025-08-01

**Authors:** Mahya Tavan, Nick W. Smith, Andrew J. Fletcher, Jeremy P. Hill, Warren C. McNabb

**Affiliations:** ^1^Sustainable Nutrition Initiative^®^, Riddet Institute, Massey University, Palmerston North, New Zealand; ^2^Fonterra Research and Development Centre, Palmerston North, New Zealand

**Keywords:** sustainable diets, nutrition, bioavailability, linear programming, dietary optimisation, nutrient adequacy

## Abstract

Over the past decade, there has been an increasing interest in the environmental sustainability of diets because food systems are responsible for a third of the anthropogenic greenhouse gas emissions (GHGE). However, less attention has been paid to the nutrient adequacy, consumer acceptability, and affordability of such diets. Such knowledge is particularly scarce in New Zealand, where approximately 40% of adults and 20% of children may live under severe to moderate food insecurity. The iOTA Model^®^ is a country-specific dietary optimisation tool designed to fill this gap by bringing the various aspects of diet sustainability together and providing evidence-based knowledge on not just the environmental impact of food but also its economic and nutritional sustainability. The iOTA Model^®^ was constructed using mixed integer linear programming by integrating New Zealand-specific dietary data. Features such as digestibility and bioavailability considerations have been incorporated as part of the iOTA Model^®^, allowing for a more accurate estimation of nutrient supply. The model is available as an open-access tool and allows users to explore various dimensions of a sustainable diet. Eight optimisation scenarios, along with baseline diets, were investigated for adult males and females in New Zealand. Results showed that reducing dietary GHGE or price by approximately 80% was possible while meeting nutrient adequacy requirements. However, such diets deviated substantially from the baseline eating patterns, indicating lower consumer acceptability, and only included a limited variety of foods. On the contrary, diets with minimum deviation from baseline remained realistic while adhering to nutrient targets and reducing GHGE by 10 and 30% in female and male consumers aged 19–30 years, respectively, and weekly price remained below the baseline. Expansion of the model to additional countries and its open-access nature will allow independent dietary sustainability research through optimisation.

## Introduction

Nutrient-adequate diets are essential for supporting human health and reducing the risk of disease. However, diets often fall short in providing adequate nutrients, and deficiencies persist worldwide across all income levels. Stevens et al. (1) estimated that two-thirds of non-pregnant women of childbearing age around the world do not meet their micronutrient requirements, with almost 50% of women and children in high-income countries estimated to have at least one micronutrient deficiency. In contrast, undernutrition can coexist with excessive intakes of energy and other nutrients, which can lead to adverse health effects such as cardiovascular disease and obesity. The global adult population affected by obesity is steadily increasing, with an additional 2.6% of individuals becoming affected each year (2).

Beyond individual health, food consumption also impacts planetary health by driving demand for the entire supply chain from farm to plate and beyond. The global food system is responsible for 34% of human-caused greenhouse gas emissions (GHGE) (3). Research by Steenson and Buttriss (4) shows that following national dietary guidelines offers approximately 20–50% lower GHGE and land use. These results are due to the common theme of higher intake of fruits and vegetables, legumes and pulses, and lower meat consumption in dietary guidelines, which are often associated with reduced environmental footprint (5). An increasing number of countries, such as Belgium, Denmark, and Qatar, are taking a step further by beginning to integrate environmental considerations directly into their food-based dietary guidelines (FBDG) (6, 7). A recent study by Trolle et al. (8) reviewed the latest Nordic and Baltic studies on the sustainability of diets and recommended a five-step methodology to integrate environmental considerations into dietary guidelines. The steps include identifying a nutritionally sound reference diet, setting environmental limits, consulting stakeholders to capture broader sustainability challenges, adapting diets to balance health and environmental objectives, and shaping these outcomes into implementable policy. For example, Denmark’s most recent FBDG, Good for Health and Climate, was informed by modelling diets with favourable health and climate profiles and was refined through stakeholder engagement to ensure practical relevance and clarity.

According to the Food and Agriculture Organization (FAO) and the World Health Organization (WHO), sustainable healthy diets are ‘dietary patterns that promote all dimensions of individuals’ health and well-being; have low environmental pressure and impact; are accessible, affordable, safe and equitable; and are culturally acceptable’ (32). This definition emphasises that sustainable, healthy diets must address all pillars of sustainability, rather than focusing on just one. Importantly, the acceptability of diets is often missing from the diet sustainability discourse, even though it is crucial in determining consumers’ willingness to adopt sustainable diets. Failing to account for diet acceptability can result in generating diets that are mostly unrealistic, even if they are environmentally friendly and even nutrient sufficient (9). Incorporating consumer acceptability into modeled diets in a meaningful way poses challenges, with the predominant approach involving minimising changes from current dietary patterns (10).

Achieving a balance between environmental sustainability, nutritional adequacy, affordability, and consumer acceptability within an entire diet context is complex and requires considering numerous variables and nuances. Dietary optimisation modelling is a powerful method in simulating diets that fulfil parameters such as nutritional requirements, environmental and economic constraints, while also considering consumers’ current eating patterns (11). The aim of optimisation modelling is to generate diets that fulfil a set of objectives and constraints and generate one or a range of solutions to the user-defined problem. In recent years, optimisation modelling has been used in generating theoretical diets that align with the various pillars of sustainable healthy diets. These models vary in their approach—some operate at the level of whole diets or food groups, while others, like The iOTA Model®, optimise at the level of individual food items. Each of these approaches has distinct strengths and limitations depending on the modelling objective, such as targeting minimal dietary shifts, enhancing nutrient adequacy, or achieving long-term environmental gains. One example is the Sustainable, Healthy, Acceptable, Realistic, and Preferable diets (SHARP) model, a tool designed to optimise observed diets of individuals for health and environmental sustainability. The SHARP model is diet based which means that it calculates linear combinations of the observed diets that meet, or are closer to, nutrient targets than the baseline diet (12). A limitation of this approach is that finding a perfect solution relies on the assumption that the ideal diet already exists among the observed individuals. This means that the rest of the population is benchmarked against existing diets, rather than having those diets adjusted to meet specific nutrient or acceptability targets. Another example is optimeal which optimises diets for nutrition and environmental impact at the individual or national level (13). Each of these models provides valuable insights in certain contexts and complements each other. It should also be noted that many of these models are not currently open-access, which can limit their broader applicability and uptake.

This study introduces a novel open-access dietary optimisation tool called The iOTA Model^®^ (iOTA hereafter) which can generate country-specific nutrient-adequate diets for different age groups and sexes. This study focuses on selected outcomes of the model for New Zealand.

## Methods

### Food database compilation

A list of 346 individual food items was adopted from a previous study by Drew et al. (14) in which the authors selected foods from the New Zealand Adult Nutrition Survey (NZANS) 2008/09 to serve as a reference list for the development of a New Zealand Food Emissions Database. Nutrient compositions of these items were obtained from New Zealand’s food composition database (FOODfiles), which comprises food composition data for over 2,700 foods. The food items of the reference dataset were matched to the entries of the FOODfiles database and their corresponding composition. Ideally, the three best-matching compositions were averaged to provide the composition for the food item on the reference list. In cases where fewer than three matches were found, the best one or two compositions were used instead. When matching food items to compositions, ‘plain’ versions were prioritised (e.g., plain pasta, not vegetable flavoured) and ‘cooked’ rather than ‘raw’ where cooking is necessary for edibility (e.g., chicken meat), but both ‘cooked’ and ‘raw’ where a food can be consumed in either form (e.g., carrots). Where nutrition survey data included more than one individual food under a single item name (e.g., the item onion/garlic/leeks), at least one composition for each particular food was included in the averaged composition for the food item, even if this meant including more than three compositions in the calculation of average composition.

In addition, where data could not be sourced from FOODfiles (e.g., individual amino acid profile), USDA Food Data Central was used as a supplementary data source. For each food item included in the dataset, a matching composition or set of compositions was identified in the USDA database, as performed for the FOODfiles. Amino acid content was then normalised to total protein content, and this normalised value was used in the model dataset. A bioavailability coefficient (ranging from 0 to 1) was applied to the total amount of proteins and individual essential amino acids to calculate the proportion of the nutrient that is absorbable (15, 16).

GHGE associated with the food items were adapted from the reference mentioned earlier (14) with minor modifications (17). This life-cycle assessment (LCA) data, which includes per-kilogram cradle to point-of-sale emissions estimates over multiple stages including farming and processing, transportation, transit packaging, consumer packaging, warehouse and distribution, refrigeration, and supermarket overheads, was compiled based on data gathered from a wide range of studies undertaken in New Zealand and elsewhere.

Retail prices of the food items were collected online from three mainstream supermarkets run by Foodstuffs Ltd. and Woolworths New Zealand Ltd., which collectively hold the vast majority of the New Zealand market share. Supermarket brands used for price collection in this study included *Woolworths*, *New World* and *Pak’n Save*.

For each supermarket website, store locations were set to one of the following cities in New Zealand, which are collectively home to approximately 52% of the national population, spanning both major islands and a range of urban densities: Auckland, Wellington, Palmerston North, and Christchurch. Prices were collected from at least one store per supermarket brand in each city (See supplementary material for a complete list of store locations), resulting in over 8,000 price datapoints. If similar items had varying prices in different stores, an average price was calculated. Supermarket items were matched to the main database by selecting the best-matching foods that represented each of the iOTA items. Where there was more than one match available, the average of the best matching items was considered.

### Baseline diets

Female and male baseline diets were adapted from the simulated typical diets developed for use in the 2016 New Zealand Total Diet Study (18). Full methodology for the development of the simulated typical diets is described elsewhere (19). Briefly, baseline diets consist of 132 food items selected based on their frequency of consumption as reported in the latest national food intake survey (NZANS 2008/9), with adjustments to include the most relevant food items and serving sizes. As the simulated typical diets were primarily developed for use in the studies concerning agricultural contaminants, they include five food items that are not frequently consumed but impose a significant risk of exposure to hazardous compounds. These five items (mussels, shrimps, prawns, oysters, and lamb’s liver) were removed from the baseline diets used in this study.

### Nutritional requirements

The Nutrient Reference Values (NRVs) for Australia and New Zealand (20) were used to determine target intakes for nutrients. The NRVs incorporate data on ‘Recommended Dietary Intakes’ (RDIs) or ‘Allowances’ which represent the ‘average daily amounts of essential nutrients necessary for sustenance or prevention of deficiency states’. Upper limits (UL) were included where available. A list of NRVs used in this study can be found in the Supplementary Table S3.

Since it is inappropriate for a dietary model to recommend more alcohol consumption than the current consumption levels, alcoholic beverages were omitted from the tested scenarios in this study. However, online users of iOTA can set their own preferences.

### Optimisation framework and scenarios

Mixed integer linear programming was used to develop optimised diets. The objective function was either to deviate as little as possible from the baseline diet (in terms of number of serving sizes), or to minimise diet price, or diet emissions, depending on the scenario. The optimisation was always constrained to deliver nutrients in line with the NRVs, while also adhering to various price and emissions constraints in some scenarios.

All analysis was carried out in R version 4.4.2 and leveraged the ompr and R Optimisation Infrastructure packages. Gurobi Optimization software (Gurobi Optimization, LLC, USA) was used to achieve faster solve times (31).

Eight optimisation scenarios, along with the baseline scenario, were investigated for each sex. These scenarios are described in Table 1.

**Table 1:** Description of scenarios used in this study.

Scenario	Description	Label
Baseline diet	Simulated baseline diet for adult males or females. This is reflective of a typical New Zealand consumption pattern as reported in the NZANS 2008 / 9.	M. Base or F. Base
Nutrient-adequate diet with minimum deviation from baseline diet	Minimum change from baseline diet to meet nutrient adequacy for 19–30-year-old men or women.	M. NAD or F. NAD
	As above, for 31–50-year old men or women.	M. NAD_35 or F. NAD_35
	As above, for 51–69-year-old men or women.	M. NAD_55 or F. NAD_55
	As above, for over 70-year-old men or women.	M. NAD_75 or F. NAD_75
Price reduced nutrient-adequate diet	Minimum change from baseline diet to meet nutrient adequacy and a 70% price cap.	M. NAD_P or F. NAD_P
GHGE reduced nutrient-adequate diet	Minimum change from baseline diet to meet nutrient adequacy and a 70% GHGE cap.	M. NAD_E or F. NAD_E
Minimum GHGE nutrient-adequate diet	The least GHGE nutrient-adequate diet. No constraint on deviation from baseline diet.	M. Min_E or F. Min_E
Minimum price of nutrient-adequate diet	The least expensive nutrient-adequate diet. No constraint on deviation from baseline.	M. Min_P or F. Min_P

Labels starting with ‘M’ and ‘F’ denote male and female population groups, respectively.

## Results

### Food groups

**Figure 1:**
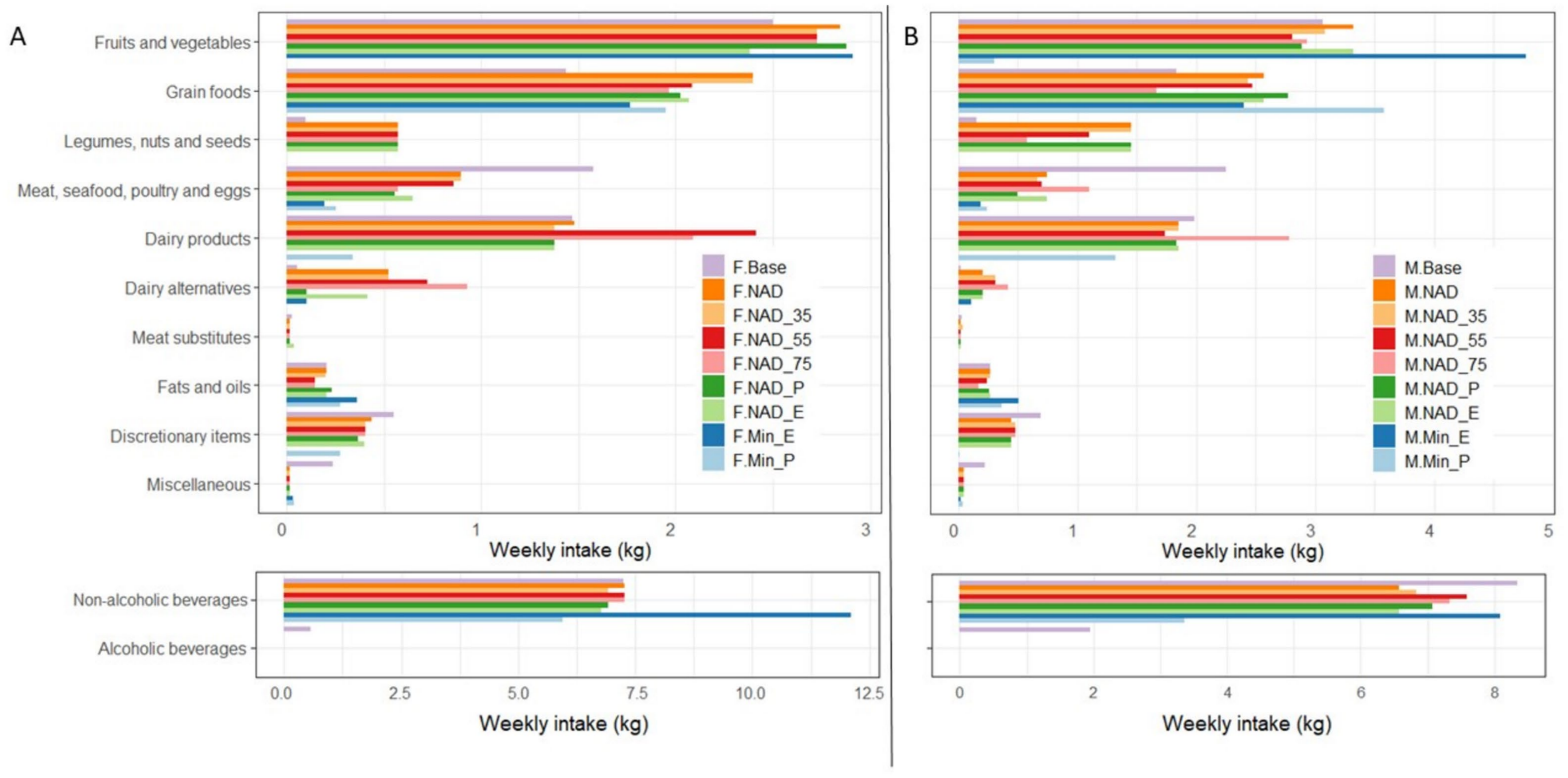
Weekly food group intake across various dietary scenarios for female **(A)** and male **(B)** consumers in New Zealand. M.Base or F.Base: Baseline diet. M.NAD or F.NAD, M.NAD_35 or F.NAD_35, M.NAD_55 or F.NAD_55, M.NAD_75 or F.NAD_75: Nutrient adequate diet with minimum deviation from baseline for the 19-30, 31-50, 5-69, and 70+ age groups, respectively. M.NAD_P or F.NAD_P: Price reduced nutrient adequate diet. M.NAD_E or F.NAD_E: GHGE reduced nutrient adequate diet. M.Min_E or F.Min_E: minimum GHGE nutrient adequate diet. M.Min_P or F.Min_P: Minimum price nutrient adequate diet. Labels beginning with ‘M’ denote males, and those starting with ‘F’ denote females. If no age group is specified, it refers to the 19-30 year age group. Alcoholic beverages are deliberately excluded in all optimised scenarios.

Weekly intake of each food group across various dietary scenarios is shown in Figure 1 and reported in detail in Supplementary Table S1. Common trends were observed in all scenarios tested across some of the broad food groups: consumption of discretionary items and meat, seafood, poultry, and eggs (MSPE) decreased in almost every scenario, while legumes, nuts and seeds, and grain foods increased overall. Changes were not as consistent for other food groups.

Baseline intake of fruits and vegetables was approximately 2,500 g per week for females and 3,000 g per week for males. In optimisation scenarios for females, fruit and vegetable intakes increased by up to 16%, except in F. NAD_E and F. Min_P scenarios, where intakes decreased by 5 and 100%, respectively. For male consumers, fruit and vegetable intakes remained similar to the baseline except in two scenarios. A 90% reduction from the baseline intake of fruits and vegetables was observed in M. Min_P, while M. Min_E showed 56% higher consumption than the baseline intakes.

Grain foods, which consist of four subgroups: (1) bread and bread products, (2) breakfast cereals, (3) flour and bran, and (4) rice, noodles, and pasta were consumed at 1,435 and 1,834 g per week in female and male baseline diets, respectively. Overall, intake of this food group increased across all scenarios; however, the contribution of the four subgroups within grain foods varied between the scenarios.

Conversely, baseline intake of legumes, nuts, and seeds was 98 and 147 g per week for female and male consumers, respectively. A notable increase of up to 573 g per week for females and up to 1,453 g per week for males was observed when minimising change from baseline diets (all scenarios with ‘NAD’ in their label). However, in F. Min_E, M. Min_E, F. Min_P, and M. Min_P scenarios, intake levels for the legumes, nuts, and seeds group dropped to 0 g per week.

Baseline consumption of MSPE was 1,575 and 2,254 g per week for female and male consumers, respectively. A notable drop in this group of food items was observed across all tested scenarios, ranging from 43% in F. NAD and F. NAD_35, to 92% in M. Min_E. Foods in the MSPE group that were selected in one or more of the optimised scenarios included but were not limited to chicken muscle meat (e.g., chicken breast), cooked eggs, fish products (e.g., fish fingers), offal (e.g., liver or kidney), and ham.

Baseline intake of dairy products was 1,470 g per week for female consumers and 1981 g per week for male consumers. Dairy consumption remained mostly stable when minimising change from baseline diets (all scenarios with ‘NAD’ in their label), and only increased in F. NAD_55, F. NAD_75, and M. NAD_75 by 64, 42, and 40%, respectively. Dairy products were significantly reduced in F. Min_P and M. Min_P scenarios by approximately 77 and 34%, respectively. No dairy products were included in F. Min_E and M. Min_E scenarios. Dairy items selected in optimised scenarios included trim, flavoured and homogenised milk, regular yoghurt, high-fat cheese, and ice cream.

Baseline intake of dairy alternatives was 56 g per week for female consumers and 21 g per week for male consumers, confined to a single product in the baseline diet used in iOTA: soy milk alternative. Notable changes in dairy alternative intakes were observed across several tested scenarios. For females, dairy alternatives ranged from 0 g per week in F. Min_P to 927 g per week in F. NAD_75. For males, intakes ranged from 0 g per week in M. Min_P to 414 g per week in M. NAD_75. Soy milk and yoghurt were the two dairy alternatives most often selected in optimised scenarios.

Discretionary foods decreased across all scenarios by at least 21% for females and 31% for males. Discretionary items consumed the most in both female and male baseline diets included cake-type desserts, milk puddings (rice pudding, instant puddings, custards, and trifle), and sugar. No discretionary items were selected in both F. Min_E and M. Min_E scenarios. In contrast, discretionary items were selected at 275 g per week in F. Min_P and 11 g per week in M. Min_P, consisting entirely of a single item: sugar.

Fats and oils results varied across the scenarios. Baseline intake of fats and oils was 203 g per week for females and 266 g per week for males, consisting of three items: monounsaturated margarine, olive oil, and butter. Two new items that were not present in the baseline diets were introduced in some scenarios: high polyunsaturated fatty acid (PUFA) oils (such as sunflower oil and soybean oil) and high monounsaturated fatty acid MUFA oils (such as canola oil and peanut oil). For both sexes, high PUFA and high MUFA oils were the only items within the fats and oils group to be selected in Min_E and Min_P scenarios, bringing the total fats and oils intakes to 361 and 274 g per week in F. Min_E and F. Min_P and 502 and 362 g per week in M. Min_E and M. Min_P, respectively.

The miscellaneous group, consisting of sauces, soups, and flavouring, is consumed at 238 and 224 g per week in female and male baseline diets, respectively. Overall, the intake of food items belonging to this group dropped across all scenarios. However, in the Min_E and Min_P scenarios for both females and males, the item condiments, salt, and flavouring were introduced to the optimised diets at quantities smaller than 40 g per week.

Non-alcoholic beverages are consumed at 7,252 and 8,344 g per week in female and male baseline diets, respectively. Intakes of non-alcoholic beverages remained largely similar to baseline when minimising change from baseline diets (all scenarios with ‘NAD’ in their label). Most notable changes in non-alcoholic beverages occurred in F. Min_E, where they increased by 67%, as well as M. Min_P, where they decreased by 60%.

### Nutrient breakdown

Weekly contribution of food groups to the adequacy of selected nutrients can be found in Supplementary Table S2.

The baseline diets showed several nutrient inadequacies. Both male and female baseline diets failed to meet the target intakes for fibre, biotin, vitamin A, vitamin K, calcium, iodine, magnesium, and polyunsaturated fatty acids (PUFA). Additionally, the female baseline diet was insufficient in folate, iron, and manganese, while the male baseline diet lacked adequate copper, potassium, zinc, and linoleic acid. In contrast, saturated fatty acids and sodium exceeded the safe upper intake levels in both male and female diets, whereas total fat intake was only excessive in the male baseline diet.

Baseline fibre intakes were 156 and 202 g per week in female and male diets, respectively. Optimised female diets showed a minimum fibre intake of 204 g per week in F. NAD_P to a maximum of 400 g per week in F. Min_P. Optimised male diets showed a minimum of 7,000 mg per week in M.NAD and M.NAD_E to a maximum of 9102 mg per week in M.NAD75. This increase was a result of the inclusion of more grain foods, legumes, nuts and seeds, and fruits and vegetables.

Baseline calcium intakes were 5,088 and 6,556 mg per week in female and male diets, respectively. Optimised female diets showed a minimum calcium intake of 7,002 mg per week in F. NAD_35 to a maximum of 9,134 mg per week in F. NAD_55. Optimised male diets showed a minimum of 7,004 mg per week to a maximum of 9,102 mg per week in M. NAD_75. The increased calcium content in optimised diets was mostly attributable to a greater contribution from grain foods, with dairy products and their alternatives also contributing in certain cases. Overall, dairy products contributed a similar amount of calcium or higher than what they contributed to the baseline diet (2411 and 3143 mg per week in F.Base and M.Base, resectively), except in Min_E scenarios where they made no contribution. Min_P scenarios showed a considerable increase in calcium contribution of dairy products (4,172 and 4,799 mg per week in F. Min_P and M. Min_P diets, respectively) while dairy alternatives made no contribution to such diets. The calcium content of optimised diets for older adults was considerably higher than in other tested scenarios. In these diets, dairy alternatives contributed 814 to 1,055 mg per week in F. NAD_55 and F. NAD_75, respectively, and 341 to 462 mg per week in M. NAD_55 and M. NAD_75, respectively. Dairy products played a larger role in meeting calcium requirements for older adults, contributing 3,925 and 3,442 mg per week in F. NAD_55 and F. NAD_75, respectively, and 2,790 and 4,569 mg per week in M. NAD_55 and M. NAD_75, respectively.

Baseline iodine intakes were 644 and 887 μg per week in female and male diets, respectively. Iodine content in optimised diets ranged from 1,050 to 1,155 μg per week in female diets and 1,050 to 1,083 μg per week in male diets. In female diets optimised for minimum deviation from baseline (all scenarios with ‘NAD’ in their labels), dairy alternatives made the largest contribution to iodine adequacy (up to 548 μg per week) except in F. NAD_P where dairy alternatives made a minimal contribution. In Min_E or Min_P for both female and male groups, condiments, salt, and flavouring notably contributed to iodine adequacy.

Baseline intakes of PUFA were 86 and 111 g per week in female and male diets, respectively. Optimised female diets showed a minimum PUFA intake of 100 g per week in F. NAD_55 to a maximum of 181 g per week in F. Min_P. Optimised male diets showed a minimum of 124 g per week in M. NAD_75 to a maximum of 208 g per week in M. Min_P. The increased PUFA content in optimised diets was mostly due to the increased contribution of grain foods, fats, and oils.

Although iron and folate intakes were below recommended levels only in female baseline diets, these nutrients increased in both female and male optimised diets. For females, weekly iron intake increased from 68 mg per week in the baseline diet to up to 185 mg per week in the F. Min_E scenario. For males, iron intake increased from 90 mg per week in the baseline diet to up to 232 mg per week in the M. Min_E scenario. The increases were primarily driven by higher contributions from grain foods, along with legumes, nuts, and seeds in most scenarios, except in M. Min_E and M. Min_P scenarios. Dairy alternatives also contributed up to 23 mg per week of iron in optimised female diets, except in F. Min_P.

Folate content in female diets increased from 2,358 μg per week in the baseline to up to 5,958 μg per week in the F. Min_E scenario. This increase was largely driven by increased contribution of grain foods, fruits and vegetables, as well as legumes, nuts, and seeds, except in F. Min_E and F. Min_P scenarios, in which case legumes, nuts, and seeds had no contribution to folate intake.

For females, the baseline diet consisted of 496 g per week of protein, while F. NAD, F. NAD_E, and F. NAD_P showed a protein content of 518, 442, and 405 g per week, respectively. F. Min_E and F. Min_P had lower protein contents at 351 and 394 g per week, respectively. For males, the baseline diet consisted of 650 g per week of protein, while M. NAD, M. NAD_E, and M. NAD_P scenarios showed a protein content of 632, 632, and 588 g per week, respectively. M. Min_E and M. Min_P scenarios showed a protein content of 458 and 595 g per week, respectively.

Optimised diets showed changes in the contribution of food groups to the protein supply. Top food groups contributing to the protein supply of baseline diets are MSPE (272 to 361 g per week), grain foods (92 to 121 g per week), and dairy products (65 to 84 g per week). In all optimised diets, protein contributions from MSPE decreased, while contributions from grain-based foods increased. The share of protein from legumes, nuts, and seeds rose in diets optimised for minimal deviation from the baseline diet but showed no contribution in Min_P and Min_E scenarios. Dairy products provided similar or higher protein content in all optimised diets, except in Min_E scenarios, where they made no contribution.

### Greenhouse gas emissions (GHGE)

**Figure 2:**
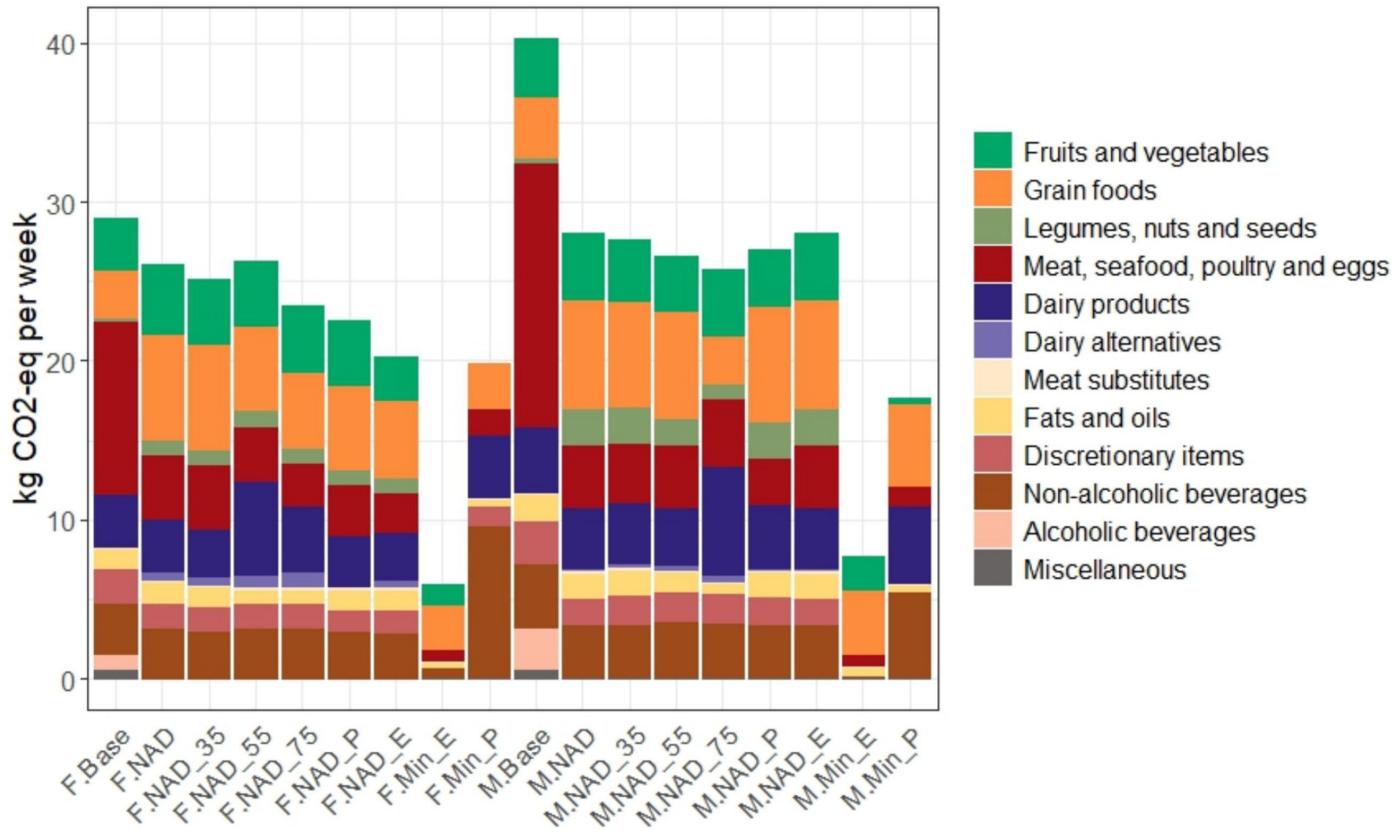
Weekly greenhouse gas emissions (GHGE) of baseline and optimised diet scenarios, colour coded by food group. Scenarios included M. Base or F. Base: Baseline diet. M. NAD or F. NAD, M. NAD_35 or F. NAD_35, M. NAD_55 or F. NAD_55, and M. NAD_75 or F. NAD_75: Nutrient-adequate diet with minimum deviation from baseline for the 19–30, 31–50, 51–69, and ≥70 age groups, respectively. M. NAD_P or F. NAD_P: Price reduced nutrient-adequate diet. M. NAD_E or F. NAD_E: GHGE reduced nutrient-adequate diet. M. Min_E or F. Min_E: Minimum GHGE nutrient-adequate diet. M. Min_P or F. Min_P: Minimum price nutrient-adequate diet. Labels beginning with ‘M’ denote males, and those starting with ‘F’ denote females. If no age group is specified, it refers to the 19–30-year age group. Alcoholic beverages are deliberately excluded in all optimised scenarios.

Weekly diet-related GHGE for all tested scenarios is shown in Figure 2. GHGE associated with the baseline diet were approximately 29 kg CO_2_-eq per week for females and 40 kg CO_2_-eq per week for males. Food groups contributing the most to the emissions associated with the female and male baseline diets were MSPE (11–16 kg CO_2_-eq per week), dairy products (3–4 kg CO_2_-eq per week), and fruits and vegetables (3–4 kg CO_2_-eq per week).

All optimised diet scenarios for both males and females had lower GHGE than the baseline diet. In F. NAD and M. NAD scenarios, GHGE dropped by approximately 10 and 30%, respectively. This reduction was mainly driven by the reduced contribution of MSPE, discretionary items and alcoholic beverages, offset by increases in the grain foods and fruits and vegetables groups. Changes in other food groups were smaller and not as consistent between scenarios. Male diets were consistently higher in emissions than female diets, but the food group pattern was similar between the sexes.

The lowest dietary GHGE in the optimised diets was approximately 6 kg CO_2_-eq per week (79.4% reduction from baseline diet), which was achieved by allowing unrestricted deviation from the baseline diet in the F. Min_E scenario. Even though all nutrient targets were met in this diet, the outcomes had very low food variety and high reliance on fortified foods.

### Price

**Figure 3:**
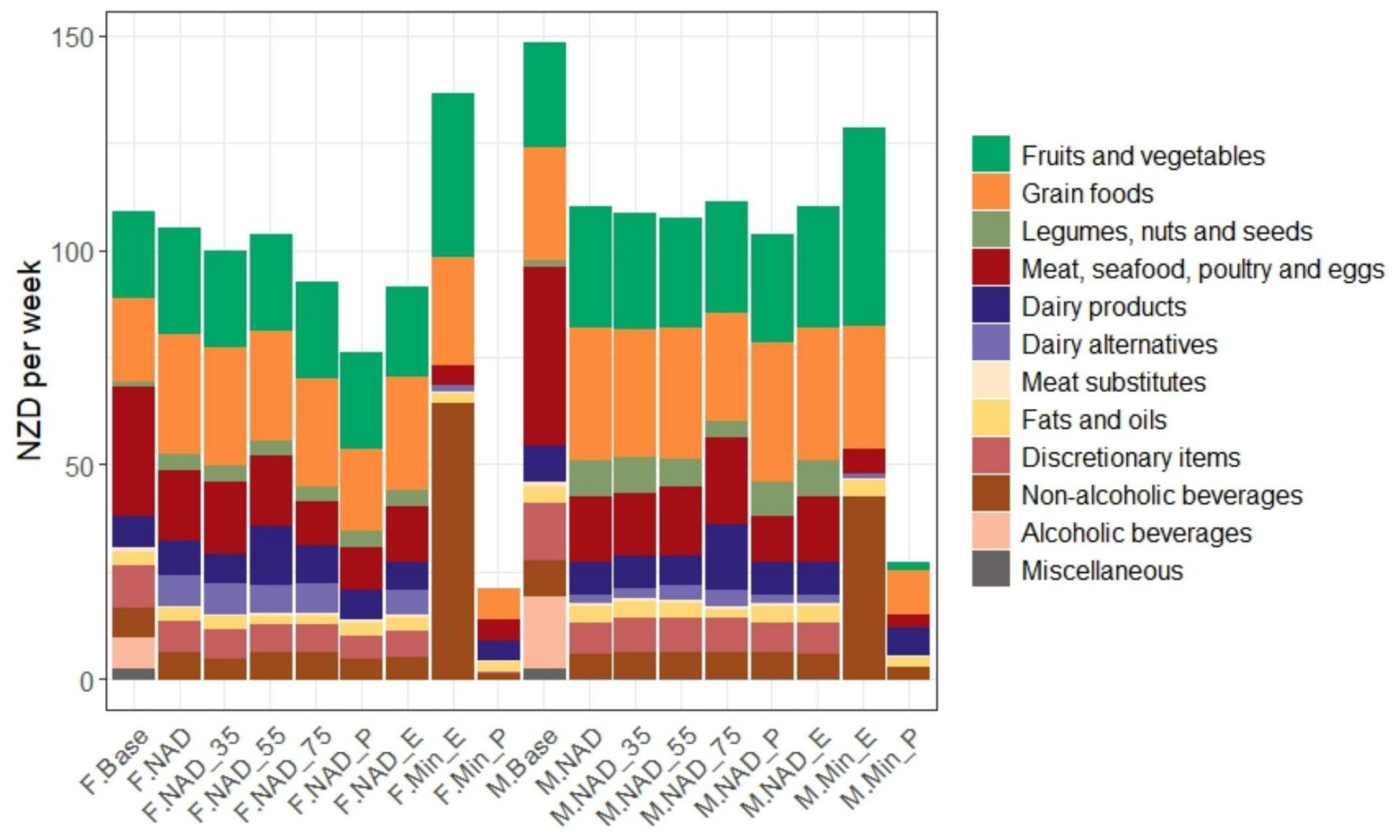
Weekly price of baseline and optimised diet scenarios, colour coded by food group. Scenarios included M. Base or F. Base: Baseline diet. M. NAD or F. NAD, M. NAD_35 or F. NAD_35, M. NAD_55 or F. NAD_55, M. NAD_75 or F. NAD_75: Nutrient-adequate diet with minimum deviation from baseline for the 19–30, 31–50, 51–69, and ≥70 age groups, respectively. M. NAD_P or F. NAD_P: Price reduced nutrient-adequate diet. M. NAD_E or F. NAD_E: GHGE reduced nutrient-adequate diet. M. Min_E or F. Min_E: Minimum GHGE nutrient-adequate diet. M. Min_P or F. Min_P: Minimum price nutrient-adequate diet. Labels beginning with ‘M’ denote males, and those starting with ‘F’ denote females. If no age group is specified, it refers to the 19–30-year age group. Alcoholic beverages are deliberately excluded in all optimised scenarios.

Weekly prices of the dietary scenarios are shown in Figure 3. The weekly price of the baseline diet was 109 NZD per week for females and 148 NZD per week for males. The lowest price of optimised diets was 21 and 27 NZD per week in F. Min_P and M. Min_P, respectively. Grain foods and dairy products were the top two food groups in these diets, collectively accounting for more than 50% of the total price.

All optimised diets for males were notably less costly than the baseline (13–82% reduction), largely driven by less spending on MSPE, alcoholic beverages, and discretionary items. For females, the reduction was modest, and an increase in price was seen in F. Min_E. Both F. Min_E and M. Min_E, showed higher prices than all other optimised diets (and higher prices than the baseline diet in the female case) at 137 and 128 NZD per week, respectively. This increase in diet price was in part due to higher dietary spending on non-alcoholic beverages (e.g., fortified drinks) as well as fruit and vegetables in the mentioned scenarios.

## Discussion

iOTA generates country-specific, nutrient-adequate diets for different age and sex groups while adhering to price and environmental constraints. It optimises diets at the level of individual food items, ensuring that realistic serving sizes of all foods are returned. This detailed approach offers a nuanced view of how each food contributes to nutritional composition, environmental footprint, and price of the diet, allowing flexibility in selecting optimal foods within food groups to meet optimisation targets. Additionally, iOTA was designed to adapt to updates and incorporate new food items as they become available in the dynamic food market landscape. In this study, we demonstrated the utility of iOTA by producing several nutrient-adequate diets meeting different criteria for various demographic groups in New Zealand. Results differed between scenarios, but overall, optimised diets had 43–51% lower MSPE, 290–485% more legumes, 9–23% more grain foods, 3–13% lower price, and 9–30% lower GHGE than baseline diets, at a minimum.

The baseline diet for adult female and male New Zealanders was associated with estimated GHGE of 26 and 40.26 kg CO_2_-eq per week, respectively, with a significant portion attributed to animal-sourced foods such as ruminant meat. In terms of nutrient adequacy, the baseline diets failed to meet recommended intakes for several nutrients such as fibre, biotin, vitamin A, calcium, copper, iodine, magnesium, zinc, PUFA, folate, and iron.

By allowing unrestricted deviations from baseline diets for males and females aged 19–30, substantial GHGE reductions of approximately 80% were achieved for both gender groups. While these diets met all nutrient targets, they were unrealistic and impractical due to their significant departure from current dietary habits and limited food variety. They heavily depended on a limited number of foods, such as fortified breakfast cereals, to reach nutrient adequacy, while excluding several food groups, such as fruits, nuts and seeds, legumes and pulses, and dairy products. This illustrates the important differences between nutrient adequacy and adhering to food-based dietary guidelines at the food group level: it is possible for a diet to meet one while not meeting the other. As shown in this study, the minimum emission (and minimum price) nutrient-adequate diets were far from the food-based dietary guidelines due to their lack of diversity and exclusion of several recommended food groups. In contrast, the nutrient adequacy of diets designed based on combinations of healthy foods has been questioned in the past (21). Incorporating food-based dietary guidelines into future iOTA work would provide an interesting case study of the differences between diets that are nutritionally adequate, meet food-based dietary guidelines, or do both simultaneously.

Unlike minimum emission and price diets, when deviation from the baseline diets was minimised, iOTA generated solutions that met all nutrient recommendations while including a notably more diverse range of food items. These diets reduced GHGE by 30% for males and 10% for females aged 19–30 years old. Consumption of discretionary items and animal-sourced foods decreased in almost every scenario, while legumes, nuts, and seeds, and grain foods increased overall. This shift broadly aligns with findings from most other dietary optimisation and scenario research, in New Zealand (14) (33) and elsewhere, which indicate that dietary patterns optimised for nutrient adequacy, environmental sustainability or cost reduction often involve reduced consumption of discretionary and animal-sourced foods, coupled with increased reliance on plant-based foods such as legumes, nuts, seeds, and whole grains, due to their affordability, nutrient density, and lower environmental impact (9, 22–25).

Dairy is often retained in dietary optimisation studies due to its nutrient density and ability to efficiently meet multiple nutritional targets within constrained dietary scenarios (9, 26). Dairy products were present in most of the scenarios tested in this study. Their contribution was most significant in optimised diets for older adults through increasing the intake of low-or reduced-fat dairy items such as skim milk and low-fat yoghurt. This observation aligns with previous studies highlighting the role of dairy products in providing essential nutrients such as calcium, vitamin D, and protein, which are particularly critical for older adults to maintain bone health, prevent osteoporosis, and support overall nutrient adequacy (27).

In several optimised diets in this study, dairy alternatives, such as soy milk alternative and soy yoghurt, were selected in quantities significantly larger than the baseline, as was also the case in an optimisation study by Mazac et al. (28). This increase seems to be due to inadequate intake of iron and iodine in baseline diets, with the dominant dairy alternatives on the New Zealand market enriched with these nutrients. However, due to data limitations, the bioavailability of these nutrients was not accounted for. It is also important to note that the baseline consumption data in this study is based on national dietary surveys conducted in 2008 / 09 and may not fully reflect dietary changes that have occurred since then. Dairy alternatives are usually more expensive than conventional dairy products due to their production costs, reliance on imports, and being branded as premium products (29). Therefore, they were not selected in minimum price diets.

The total price of the baseline diets for adult females and males was 109 and 148 NZD per week, respectively. Most optimised diets had lower total prices, mainly due to the reductions in often costly items belonging to the meat, seafood, and poultry, discretionary items, and alcoholic beverages groups. Although less realistic, it was possible to bring the total price of the weekly diets down by 81–82% and still meet the nutrient adequacy targets. As also reported by Chungchunlam et al. (30), low-cost diets included a variety of animal-sourced foods, such as dairy, canned seafood, offal, and poultry, due to the high nutrient density of these foods relative to their cost.

An interesting point to note is the role of inexpensive soft drinks (with added vitamin C) in shaping the minimum price diets. These drinks, which were absent from both baseline diets, provide nearly all the required vitamin C (a vitamin typically sourced from fresh fruits and vegetables) in the minimum price diets while making up only 5% of the weekly diet cost for females and 9% for males. Conversely, no fruits and vegetables were selected in the female minimum price diet, and only a small amount was included in the male version. If soft drinks were excluded from the optimisation, a slightly smaller price reduction at 78% for females and 80% for males could be achieved while allowing fruit and vegetable intake to increase to 708.5 g per week for females and 945 g per week for males (data not shown). These changes would allocate approximately 20% of the weekly diet cost to fruits and vegetables for both groups. This highlights the trade-off between reducing costs and incorporating more nutrient-dense, health-promoting foods, emphasising the need to carefully consider the nuances when interpreting optimisation outcomes beyond nutrient adequacy.

An important caveat that must be considered when optimising diets based on food items is that food items within a food group are not necessarily devoid of ingredients from other food groups. For example, in our study, the optimised diets showed an overall increase in grain foods, a group often associated with plant-based foods. However, upon closer examination, the items driving this increase were predominantly ‘plain pasta’, ‘noodles’, and ‘fried rice/risotto/pilaff/rice salad/sushi’, many of which may contain animal-sourced ingredients, such as eggs used in making plain pasta dough or seafood used in sushi. This explains how these diets achieved the vitamin B12 requirements, an essential micronutrient naturally absent from plant-based foods, even when the food group MSPE is excluded from the optimised diet.

Despite being a powerful open-access tool for simulating various nutrient-adequate diets, iOTA has limitations. Although iOTA produces solutions that are in alignment with national nutrient reference values, it is not intended to be directly used as dietary advice for individuals or to replace the services of qualified nutrition professionals (especially where there are medical constraints that limit food choices). Besides, optimising diets at the level of individual food items can sometimes lead to impractical outcomes. As shown in the minimum price or emission scenarios in this study, when extreme settings are used, the optimisation process may generate diets that meet these constraints but include only a small number of food items, making them unrealistic and unacceptable for consumers. Proximity to the observed dietary intakes of a population is often used as a proxy for quantifying the acceptability of any proposed diet, under the assumption that a population’s current eating habits are the most acceptable (10). However, it can be argued that the average intake may not fully represent the diverse eating patterns followed by individuals. This is also a limitation when estimating the baseline nutrient intakes and environmental impacts of current diets based on the population average, as an average could be driven by a wide distribution. It would be possible to incorporate ranges of current intake as model constraints in future research, as performed elsewhere (12).

## Conclusion

iOTA offers an open-access platform for optimising diets based on nutrient adequacy, acceptability, environmental impact, and price. It is intended to be used by experts in food and nutrition research and education, and to support informed decision-making.

This study demonstrated exemplary scenarios for optimising diets for adult female and male consumers across different age groups in New Zealand. The findings revealed that while various nutrient-adequate diets can be generated depending on optimisation targets and priorities, achieving diets with substantial (>50%) reductions in GHGE and price, though possible, is likely not feasible. Incorporating current dietary habits plays a vital role in designing diets that not only meet consumers’ nutritional requirements but also align with the broader pillars of sustainable healthy diets and can still achieve important reductions in GHGE (10–30%) and price (3–26%).

This study serves as a starting point to highlight the capabilities of iOTA. With its flexible and expandable design and open-access user interface, iOTA can be applied in any country to generate valuable insights into the nutritional adequacy, environmental sustainability, and affordability of diets, provided that reliable data can be obtained locally. However, further research is needed to deepen our understanding of several key factors, including the impact of specific food item selections, the role of fortified foods, and the integration of novel food products into diets. Additionally, assessing the environmental impact of diets beyond GHGE is essential, contingent on the availability of relevant data. While this study accounted for the bioavailability of proteins and amino acids, future research must also adjust for the bioavailability of critical nutrients like iron, zinc, and calcium, based on their dietary sources, to provide more accurate estimates of absorbable nutrients.

## Data Availability

The original contributions presented in the study are included in the article/Supplementary material, further inquiries can be directed to the corresponding author.
